# Sequential forward and reverse transport of the Na^+^ Ca^2+^ exchanger generates Ca^2+^ oscillations within mitochondria

**DOI:** 10.1038/s41467-017-02638-2

**Published:** 2018-01-11

**Authors:** Krishna Samanta, Gary R. Mirams, Anant B. Parekh

**Affiliations:** 1 Department of Physiology, Anatomy and Genetics, Oxford University, Parks Road, Oxford, OX1 3PT UK; 2 Centre for Mathematical Medicine and Biology, School of Mathematical Sciences, Nottingham University, Nottingham, NG7 2RD UK

## Abstract

Mitochondrial Ca^2+^ homoeostasis regulates aerobic metabolism and cell survival. Ca^2+^ flux into mitochondria is mediated by the mitochondrial calcium uniporter (MCU) channel whereas Ca^2+^ export is often through an electrogenic Na^+^–Ca^2+^ exchanger. Here, we report remarkable functional versatility in mitochondrial Na^+^–Ca^2+^ exchange under conditions where mitochondria are depolarised. Following physiological stimulation of cell-surface receptors, mitochondrial Na^+^–Ca^2+^ exchange initially operates in reverse mode, transporting cytosolic Ca^2+^ into the matrix. As matrix Ca^2+^ rises, the exchanger reverts to its forward mode state, extruding Ca^2+^. Transitions between reverse and forward modes generate repetitive oscillations in matrix Ca^2+^. We further show that reverse mode Na^+^–Ca^2+^ activity is regulated by the mitochondrial fusion protein mitofusin 2. Our results demonstrate that reversible switching between transport modes of an ion exchanger molecule generates functionally relevant oscillations in the levels of the universal Ca^2+^ messenger within an organelle.

## Abstract (web summary)

Mitochondrial Ca^2+^ homoeostasis is tightly regulated and export of Ca^2+^ is mediated by an Na^+^Ca^2+^ exchanger. Here authors show that in depolarised mitochondria the exchanger initially operates in reverse mode, transporting cytosolic Ca^2+^ into the matrix before it reverts to its forward mode state.

## Introduction

Mitochondrial Ca^2+^ import shapes the pattern of cytosolic Ca^2+^ signals and regulates ATP production and cell survival^1^. The porous outer mitochondrial membrane is freely permeable to Ca^2+^ but the inner membrane is not and therefore requires transporters to shuttle Ca^2+^ between the cytosol and mitochondrial matrix^2^. A major route for mitochondrial Ca^2+^ uptake is through the mitochondrial Ca^2+^ uniporter (MCU), a highly Ca^2+^-selective low conductance ion channel^3,4^. MCU is part of a larger complex involving regulators MICU1 and MICU2, MCUR1 and EMRE^5^. Flux through the MCU complex is determined by the prevailing electrochemical Ca^2+^ gradient^6^, with a major factor being the large electrical driving force that arises from the negative potential (~−200 mV) across the inner mitochondrial membrane.

Ca^2+^ transporters that extrude Ca^2+^ from the matrix have also been characterised at a molecular level and include Letm1 (leucine zipper-EF-hand-containing transmembrane protein 1)^7^ and mitochondrial Na^+^–Ca^2+^ exchange (NCLX)^8^. Letm1 is a Ca^2+^/2H^+^ electroneutral antiporter whereas NCLX is thought to be electrogenic^9^, although the precise Na^+^:Ca^2+^ stoichiometry is unclear^10^. In one study where the relative contributions of Letm1 and NCLX to mitochondrial Ca^2+^ export was investigated, NCLX was found to play the dominant role^11^.

Mitochondria are dynamic organelles, undergoing fusion and fission with the capacity to form reticular networks^12^. The precise architecture of mitochondria is important for cell viability, growth, proliferation and signalling^13^. Mitochondrial fusion is regulated by dynamin-related protein (Drp1) where outer and inner mitochondrial membrane fusion depend on mitofusin 1 and mitofusin 2, and OPA1, respectively^12^. Mitofusin 2 is also found on the endoplasmic/sarcoplasmic reticulum surface and is therefore thought to act as a physical tether bringing mitochondria and endoplasmic/sarcoplasmic reticulum together at specialised regions called mitochondrial associated membranes^14^. Close apposition of the two organelles allows for rapid and effective local Ca^2+^ signalling^15,16^. Ca^2+^ release from the endoplasmic reticulum by the Ca^2+^-releasing second messenger inositol trisphosphate (InsP_3_) leads to a high local Ca^2+^ signal that can be transported into mitochondria by the MCU. The rise in matrix Ca^2+^ stimulates rate-limiting enzymes in the Krebs cycle^17^, resulting in accelerated ATP production. In the heart, for example, mitochondrial fusion dynamics depends on contractile activity^18^. In cardiac myocytes, shuttling of Ca^2+^ released from the sarcoplasmic reticulum by ryanodine receptors into mitochondria drives rapid bioenergetic responses that are important for cardiac function^19^.

Stimulation of Gq protein-coupled receptors activate phospholipase C to generate InsP_3 _(ref. ^20^). Low concentrations of agonist, which are thought to mimic physiologically relevant doses, typically evoke oscillations in cytosolic Ca^2+^. The oscillations arise from regenerative Ca^2+^ release from the endoplasmic reticulum by InsP_3_-gated Ca^2+^ channels followed by store-operated Ca^2+^ entry^20^. Previous work has shown cytosolic Ca^2+^ oscillations following stimulation of native cysteinyl leukotriene type I receptors in mast cells with the natural agonist leukotriene C_4_ are propagated rapidly and faithfully into mitochondria to generate oscillations in matrix Ca^2+^ (ref. ^21^). Knockdown of the MCU or mitochondrial depolarisation, which impairs Ca^2+^ flux through the MCU, suppressed mitochondrial Ca^2+^ uptake^21^. Loss of mitochondrial Ca^2+^ buffering resulted in rundown of cytosolic Ca^2+^ oscillations, which arose through enhanced Ca^2+^-dependent inactivation of InsP_3_ receptors.

Cytosolic Ca^2+^ oscillations are sustained by Ca^2+^ entry through store-operated Ca^2+^ channels, which refill the endoplasmic reticulum with Ca^2+^ following InsP_3_-evoked Ca^2+^ release^20^. In mast cells and T lymphocytes, mitochondrial Ca^2+^ uptake sustains Ca^2+^ entry by reducing Ca^2+^-dependent slow inactivation of the store-operated Ca^2+^ channels^22,23^. In addition, mitochondria also regulate the redistribution of STIM1, a molecule necessary for the activation of store-operated Ca^2+^ channels, from the endoplasmic reticulum to the plasma membrane^24^. In mast cells, inhibition of store-operated Ca^2+^ influx following mitochondrial depolarisation can be rescued by knockdown of the mitochondrial fusion protein mitofusin 2 (ref. ^24^). In this study, we show that mitochondrial Ca^2+^ oscillations induced by leukotriene receptor stimulation that are lost following mitochondrial depolarisation can be rescued when mitofusin 2 levels are reduced. We find that the mitochondrial Ca^2+^ oscillations under these conditions arise from mitochondrial Na^+^–Ca^2+^ exchange, operating sequentially in reverse and forward transport modes. Our data show that the same transport molecule can both raise and lower matrix Ca^2+^ in response to receptor stimulation. More generally, our results reveal that an ion transporter can compensate for impaired activity of an ion channel and thereby sustain functionally relevant Ca^2+^ signals within an organelle.

## Results

### Mitochondrial depolarisation impairs Ca^2+^ signals to agonist

**Fig. 1:**
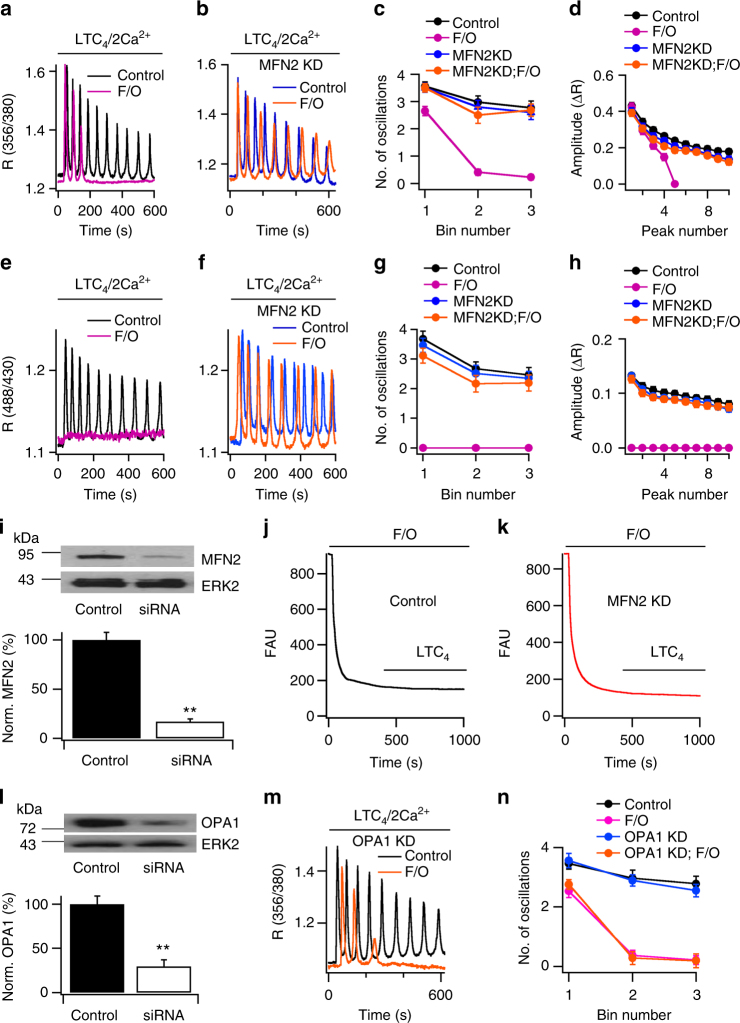
Mitofusin 2 knockdown rescues oscillatory Ca^2+^ signals in cells with de-energised mitochondria. **a** Cytosolic Ca^2+^ oscillations (measured with fura 2) to LTC_4_ (120 nM) are suppressed by FCCP (2 μM) and oligomycin (0.5 μg per ml; depicted as F/O), pre-treated for 5–10 min. **b** Mitofusin 2 knockdown has little effect on cytosolic Ca^2+^ oscillations in a cell with energised mitochondria but sustains the response in a cell pre-treated with FCCP and oligomycin. **c** The number of oscillations in each 200 s bin (measured from the application of LTC_4_) are compared for the conditions shown. Each point is the mean of 15–20 cells from three independent experiments. Control trace (black) is offset upwards by 0.1, to resolve it from other traces. **d** Oscillation amplitude is plotted against oscillation number. A peak number of 4 denotes the fourth oscillation. Control trace (black) is offset upwards by 0.04. **e** Matrix Ca^2+^ oscillations measured with the mitochondrially targeted pericam are shown for a control cell challenged with LTC_4_ and for one pre-treated with FCCP and oligomycin. **f** Matrix Ca^2+^ oscillations are compared in the absence and presence of FCCP, following mitofusin 2 knockdown. **g**, **h** The number of matrix Ca^2+^ oscillations (**g**) and the amplitude of each oscillation (**h**) are compared for the conditions shown. Each point is the mean of >14 cells from three independent experiments. In panel **g**, the control trace (black) is offset upwards by 0.1. **i** Western blot compares mitofusin 2 expression in control cells and after siRNA-targeted knockdown. The histogram summarises aggregate data from two separate samples. **j**, **k** Mitochondrial membrane potential, measured with TMRE, is shown following exposure first to FCCP and oligomycin and then LTC_4_ in a control cell (**j**) and in one following mitofusin 2 knockdown (**k**). FAU denotes fluorescence, arbitrary units. **l** Western blot compares OPA1 protein expression in control cells and after siRNA-targeted knockdown. **m** OPA1 knockdown does not rescue cytosolic Ca^2+^ oscillations in FCCP-treated cells. **n** Number of Ca^2+^ oscillations per 200 s bin are compared for the conditions shown. All data are from RBL-1 mast cells. **p < 0.01 (unpaired Student’s *t*-test). Error bars denote SEM

Stimulation of native G-protein-coupled cysteinyl leukotriene type I in RBL-1 mast cells receptors with the agonist leukotriene C_4_ (LTC_4_) increases the levels of the second messenger InsP_3_, which triggers oscillations in cytosolic Ca^2+^ (Fig. 1a)^25^. The oscillations decrease somewhat in number (Fig. 1c) and amplitude (Fig. 1d) over a 600 s period, due to receptor desensitisation^26^. Measurements of matrix Ca^2+^, using ratiometric pericam that is genetically targeted to the mitochondrial matrix, revealed oscillations in matrix Ca^2+^ following LTC_4_ challenge (Fig. 1e, g, h) that closely mirrored those in the cytosol (Fig. 1a). Mitochondrial depolarisation with the protonophore carbonilcyanide p-triflouromethoxyphenylhydrazone (FCCP) abolished prolonged oscillatory Ca^2+^ signals both in the cytosol (Fig. 1a, c, d) and mitochondrial matrix (Fig. 1e, g, h)^21^. Similar results were obtained after knockdown of the MCU^21^.

### Knockdown of Mitofusin 2 rescues matrix Ca^2+^ signals

siRNA-directed knockdown of mitofusin 2, a dynamin-related GTPase protein involved in mitochondrial fusion^27,28^, had no effect on LTC_4_-evoked oscillations in cytosolic (Fig. 1b–d) or matrix Ca^2+^ (Fig. 1f–h) in cells with an intact mitochondrial membrane potential. However, knockdown of mitofusin 2 fully prevented the loss of cytosolic (Fig. 1b–d) and matrix Ca^2+^ oscillations (Fig. 1f–h) that occurred after mitochondrial depolarisation with FCCP and oligomycin. Western blots confirmed that knockdown of MFN2 led to a significant decrease in protein levels of ~80% (Fig. 1i). Qualitatively similar results were obtained in HEK293 cells expressing the cysteinyl leukotriene type I receptor (Supplementary Figures 1 and 2).

We considered the possibility that the mitochondrial membrane potential repolarised in the presence of FCCP when leukotriene receptors were activated in mitofusin 2-deficient cells. However, this was not the case. Following treatment with FCCP and oligomycin, the potential (measured with TMRE) depolarised and remained low after stimulation with LTC_4_ for the duration of agonist exposure, both in the presence of mitofusin 2 (control, Fig. 1j) or after knockdown (Fig. 1k).

Mitofusin 2 plays an important role in regulating mitochondrial fusion^29^. However, the rescue of cytosolic Ca^2+^ oscillations that was achieved by knockdown of mitofusin 2 in cells with depolarised mitochondria (Fig. 1b–d) was not mimicked by knockdown of another fusion protein, Optic atrophy 1 (OPA1^13^ (Fig. 1l, n). Hence the involvement of mitofusin 2 on matrix Ca^2+^ is distinct from its well-documented role in mitochondrial fusion.

### NCLX rescues matrix Ca^2+^ signals in depolarised mitochondria

**Fig. 2:**
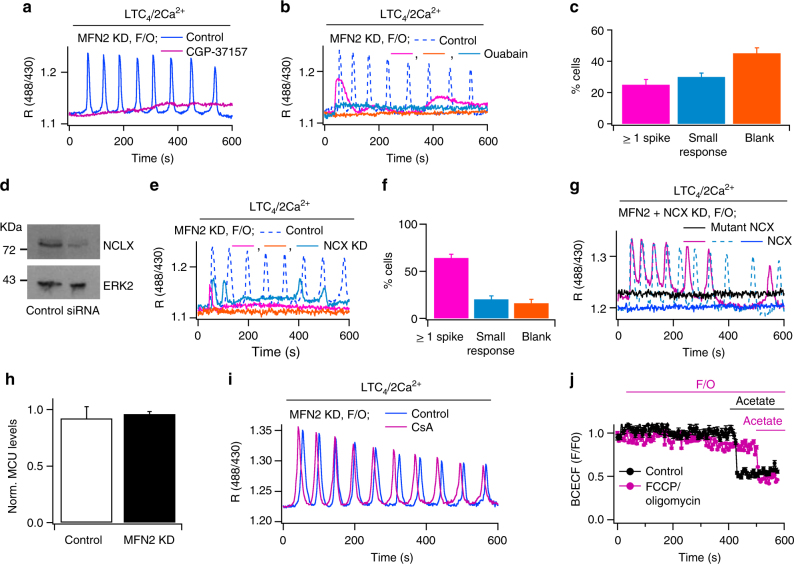
Oscillations in matrix Ca^2+^ by mitochondrial Na^+^–Ca^2+^ exchange. In panels **a**–**f**, mitofusin 2 was knocked down and cells were pre-treated acutely with FCCP and oligomycin prior to LTC_4_ challenge. Matrix Ca^2+^ was measured with ratiometric pericam. **a** Pre-exposure (10 min) to CGP-37157 (10 μM) abolished matrix Ca^2+^ oscillations to LTC_4_ (control: blue trace, typical of 21/29 cells; CGP-treated: red trace, typical of 12/12 cells). **b** As in panel **a**, but ouabain (25 μM; 15 min pre-treatment) was used to raise cytosolic Na^+^. Three types of matrix Ca^2+^ response to LTC_4_ were now observed; blank/no response (orange), a small slow rise (blue) or at least one spike (magenta). **c** Bar plot shows the fractional responses, as in panel **b**. Total number of cells analysed was 24. **d** Western blot compares Na^+^–Ca^2+^ exchanger expression in control cells and in cells after siRNA-directed knockdown. **e** Ca^2+^ oscillations to LTC_4_ are suppressed following Na^+^–Ca^2+^ exchanger knockdown. Three types of response were now observed (see panel **f**). **f** Bar plot shows the types of responses of matrix Ca^2+^ to LTC_4_ following exchanger knockdown (as described in panel **c**). Total number of cells analysed was 20. **g** Matrix Ca^2+^ oscillations to LTC_4_ were induced in control cells but not in those expressing a catalytic mutant of the exchanger. Two types of response were seen when the wild type exchanger was overexpressed: no response (9/20 cells) and oscillatory responses (11/20 cells). The two oscillatory responses (dashed blue and magenta traces) depict the two patterns of oscillatory response. **h** Histogram compares MCU expression before and after mitofusin 2 had been knocked down. **i** Matrix Ca^2+^ oscillations to LTC_4_ are compared between a control cell and one pre-exposed (15 min) to cyclosporine A. In this experiment, mitofusin 2 was knocked down and FCCP/oligomycin applied 8 min before stimulation. **j** Cytosolic pH, measured with BCECF, is compared for control cells (black trace) and cells acutely exposed to FCCP/oligomycin (as indicated; red trace). Acetate was applied at the end of the experiment to induce cytosolic acidification. Each trace is the mean of between 16 and 21 cells. Error bars denote SEM

Ca^2+^ flux through MCU is steeply dependent on the voltage gradient across the inner mitochondrial membrane^6^ and MCU open probability decreases as the membrane potential depolarises^6^. Depolarisation with FCCP almost fully suppressed mitochondrial Ca^2+^ uptake following receptor stimulation (Fig. 1e), demonstrating little flux through the MCU. The findings that the amplitude and frequency of mitochondrial Ca^2+^ oscillations induced by leukotriene receptor stimulation were similar between control cells, where Ca^2+^ entry into the matrix is provided by the MCU, and in mitofusin 2-deficient cells with depolarised mitochondria, where flux through the MCU had been compromised (Fig. 1g, h), suggest an alterative Ca^2+^ influx pathway operates under conditions of a depolarised mitochondrial membrane potential. A major route for Ca^2+^ efflux from the matrix is through electrogenic Na^+^–Ca^2+^ exchange in the inner mitochondrial membrane. In energised mitochondria and with resting cytosolic and matrix Ca^2+^ and Na^+^ concentrations, the exchanger operates in forward mode, exporting Ca^2+^ from the matrix in exchange for cytosolic Na^+^. However, in depolarised mitochondria, the exchanger switches to reverse mode importing Ca^2+^ into and extruding Na^+^ from the matrix^9.^ We designed experiments to test whether reverse mode Na^+^–Ca^2+^ exchange provided a route for mitochondrial Ca^2+^ uptake following receptor stimulation in cells with depolarised mitochondria. After knockdown of mitofusin 2, cells were treated acutely with FCCP and oligomycin and then leukotriene receptors were activated while matrix or cytosolic Ca^2+^ was measured. Agonist-evoked cytosolic Ca^2+^ oscillations (Fig. 1b–d) were rapidly propagated into the mitochondrial matrix (Fig. 1f–h). Three independent lines of evidence suggest that mitochondrial Na^+^–Ca^2+^ exchange is the main route for Ca^2+^ import under these depolarised conditions. First, inhibition of the mitochondrial Na^+^–Ca^2+^ exchanger with the benzothiazepine CGP-37157 abolished mitochondrial Ca^2+^ uptake following leukotriene receptor activation (Fig. 2a). Second, elevation of cytosolic Na^+^ concentration should reduce the trans-mitochondrial Na^+^ gradient and this will decrease reverse mode Na^+^–Ca^2+^ exchange activity. We raised cytosolic Na^+^ levels by incubating cells with ouabain, an inhibitor of the plasma membrane Na^+^–K^+^ ATPase pump. In the presence of ouabain, mitochondrial Ca^2+^ oscillations were abolished following stimulation with LTC_4_ in mitofusin 2-deficient cells with depolarised mitochondria (Fig. 2b). Three patterns of response were observed after ouabain treatment (Fig. 2b, c): some cells (45%) failed to respond at all to LTC_4_, others did so by giving a modest rise in matrix Ca^2+^ (30%) and some responded by generating 1 or more small matrix Ca^2+^ spikes (25%). Ouabain had no inhibitory effect when cells were stimulated with agonist in the absence of FCCP and oligomycin (Supplementary Figure 3). Thirdly, we used an siRNA approach to knock down the recently discovered mitochondrial Na^+^–Ca^2+^ exchanger^8^. Significant knockdown of the exchanger was seen in western blots (Fig. 2d; knockdown was 68.3 ± 7.2% in two independent experiments). In all siRNA-treated cells, mitochondrial Ca^2+^ uptake was reduced but to differing extents (Fig. 2e). In some cells (labelled blank in Fig. 2f), Ca^2+^ import was almost completely suppressed. In others (labelled small response in Fig. 2f), a single matrix Ca^2+^ spike occurred whereas in a third group (labelled >1 spike), a couple of small Ca^2+^ oscillations developed followed by a quiescent period and then one or two oscillations reappeared several tens of seconds later (Fig. 2e, f). Despite the variability, each group was very different from the corresponding control, which showed repetitive Ca^2+^ oscillations for several tens of seconds. Collectively, these experiments reveal a major role for mitochondrial Na^+^–Ca^2+^ exchange in driving oscillations in matrix Ca^2+^ following stimulation of cell-surface receptors.

To see whether the oscillations in matrix Ca^2+^ indeed reflected transport through Na^+^–Ca^2+^ exchange, we expressed a catalytically inactive mutant in which threonine 468 was replaced by serine^8^. In these experiments, endogenous exchanger protein was first knocked down using siRNA and then either wild type or mutant exchanger plasmid transfected 24 h later. Matrix Ca^2+^ oscillations to LTC_4_ stimulation were seen in ~50% (11/20) of FCCP-treated cells overexpressing Na^+^–Ca^2+^ exchange but no responses (21/21 cells) were observed when the catalytic mutant was expressed instead (Fig. 2g).

We considered other possibilities that could account for the increased mitochondrial Ca^2+^ uptake in FCCP-treated cells lacking mitofusin 2. In mitofusin 2-knock out mouse embryonic fibroblasts, MCU levels decrease by ~50%^30^. In HEK293 cells, transient knockdown of mitofusin 2 had no significant effect on MCU expression (Fig. 2h; measured using quantitative PCR 24 h after mitousin 2 knockdown), suggesting alterations in MCU expression are unlikely to explain the rescue of matrix Ca^2+^ signals in depolarised mitochondria. Dissipation of the mitochondrial potential can open the large conductance permeability transition pore, providing a route for Ca^2+^ transport across the inner mitochondrial membrane. However, the permeability transition pore inhibitor cyclosporine A failed to affect agonist-evoked oscillations in matrix Ca^2+^ in mitofusin 2-deficient cells in the presence of FCCP (Fig. 2i; 10.3 ± 0.2 oscillations were generated over 600 s in control cells and the corresponding value in cyclosporine A-treated cells was 10.1 ± 0.2). FCCP and oligomycin treatment could lower cytosolic pH and this might explain why agonist-evoked Ca^2+^ signals are impaired. However, FCCP and oligomycin had little effect on cytosolic pH (Fig. 2j).

### Functional coupling between MCU and NCLX

**Fig. 3:**
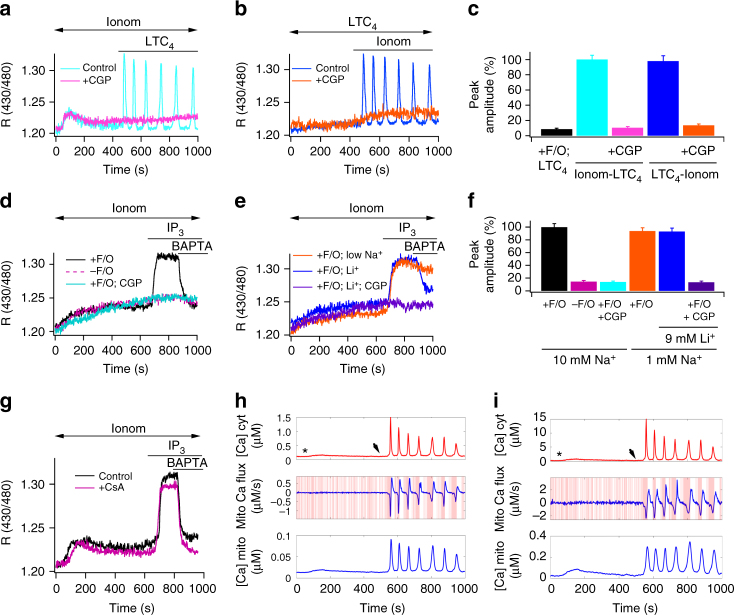
Forward and reverse mode Na^+^–Ca^2+^ exchanger. **a** After knockdown of MCU and MFN2, matrix Ca^2+^ oscillations are induced by LTC_4_ in FCCP-treated cells following a small rise in matrix Ca^2+^ by 2 nM ionomycin. Oscillations were seen in 15/24 cells. CGP-37157 suppressed this response (15/15 cells). **b** As in panel **a** but LTC_4_ is now applied first. Oscillations were seen in 10/15 cells. CGP-37157 blocked the response in 18/18 cells. **c** Aggregate data are compared. All groups were pre-treated with FCCP and oligomycin and both MCU and MFN2 were knocked down. Ionom-LTC_4_ denotes ionomycin followed by LTC_4_ (as in panel **a**). LTC_4_-Ionom denotes LTC_4_ then ionomycin, as in panel **b**. **d** Matrix Ca^2+^ is measured in permeabilised cells following stimulation with InsP_3_. Cytosolic Na^+^ was 10 mM and cytosolic Ca^2+^ was weakly buffered at 200 nM. Ionomycin was applied to raise matrix Ca^2+^, then InsP_3_ was added. F/O denotes FCCP/oligomycin. BAPTA was perfused to rapidly reduce cytosolic Ca^2+^. **e** As in panel **d**, but cytosolic Na^+^ was 1 mM. In the experiments with Li^+^, 9 mM Li^+^ was added to the 1 mM Na^+^ solution. **f** Aggregate data from experiments as in panels **d** and **e** are compared. Each bar denotes between 15 and 23 cells. **g** Ca^2+^ signals in permeabilised cells are compared in the absence and presence of cyclosporine A (1 μM). Here, mitofusin 2 had been knocked down and FCCP/oligomycin added 5–10 min before simulation with InsP_3_. Control trace is in the absence of cyclosporine A. For both traces, 1 μM ruthenium red was present throughout. **h** Simulations of Na^+^–Ca^2+^ exchanger flux (middle panel, with pink highlighted background showing where NCX is operating in reverse mode) and matrix free Ca^2+^ (bottom panel) following a cytosolic Ca^2+^ rise (upper panel) obtained from fura 2-loaded intact cells. Cells were exposed to 2 nM ionomycin (asterisk) before LTC_4_ challenge (marked by arrow; same protocol as in panel **a**). The cytosolic Ca^2+^ concentration indicated is the measured bulk concentration. **i** As in panel **h** but cytosolic Ca^2+^ has been estimated to reach 15 μM (see text). Error bars denote SEM

We asked whether LTC_4_-induced matrix Ca^2+^ oscillations in FCCP-treated cells lacking mitofusin 2 were totally independent of the MCU. Following knock down of both the MCU and mitofusin 2, stimulation with agonist in cells with a depolarised mitochondrial membrane potential now consistently failed to generate oscillations in matrix Ca^2+^ (Supplementary Figure 4). We hypothesised that, despite providing an exceedingly small Ca^2+^ flux into the matrix under the depolarised conditions that occur in the presence of FCCP^6^, the MCU nevertheless provided either trigger or facilitory matrix Ca^2+^ for driving Na^+^–Ca^2+^ exchange activity. To test this, we raised matrix Ca^2+^ very slightly by applying a low dose of the Ca^2+^ ionophore ionomycin (2 nM) to intact cells. In cells in which we reduced both MCU and mitofusin 2 expression and then depolarised mitochondria, oscillations in matrix Ca^2+^ to leukotriene receptor stimulation were rescued only if matrix Ca^2+^ had been increased slightly by ionomycin prior to agonist exposure (Fig. 3a; aggregate data are summarised in Fig. 3c). The small increase in matrix Ca^2+^ induced by ionomycin per se did not trigger Ca^2+^ oscillations (Fig. 3a). The oscillations in matrix Ca^2+^ were suppressed by CGP-37157, confirming they were mediated through Na^+^–Ca^2+^ exchange (Fig. 3a). Although LTC_4_ failed to elicit matrix Ca^2+^ oscillations in MCU-deficient cells, subsequent application of ionomycin rescued the oscillatory response and this was blocked by CGP-37157 (Fig. 3b; aggregate data are summarised in Fig. 3c).

### NCLX activity in permeabilised cells

To trap mitochondrial Na^+^–Ca^2+^ exchange in forward or reverse modes, we clamped cytosolic Na^+^ and Ca^2+^ at fixed concentrations using digitonin-permeabilised cells^9^. Ca^2+^ released from the endoplasmic reticulum by application of exogenous InsP_3_ in permeabilised RBL cells is rapidly taken up into mitochondria by the MCU^31,32^. In control cells with 10 mM cytosolic Na^+^, mitochondria sequestrated Ca^2+^ that had been released by InsP_3_ to produce a sustained elevation in matrix Ca^2+^ and this was prevented by pre-treatment with FCCP and oligomycin (Supplementary Figure 5). The matrix Ca^2+^ rise in response to InsP_3_ challenge was also prevented by pre-incubation with the InsP_3_ receptor antagonist heparin or if stores had been depleted of Ca^2+^ by prior exposure to thapsigargin (Supplementary Figure 5), demonstrating the matrix Ca^2+^ rise is due to InsP_3_-dependent Ca^2+^ release from the endoplasmic reticulum. After knockdown of MCU and MFN2, we raised matrix Ca^2+^ slightly by exposure to 2 nM ionomycin and then applied InsP_3_. InsP_3_ now failed to raise matrix Ca^2+^ (red trace in Fig. 3d; aggregate data in Fig. 3f). However, if mitochondria were depolarised by exposure to FCCP in MCU- and mitofusin 2-deficient cells, matrix Ca^2+^ increased following InsP_3_ stimulation (Fig. 3d, f), indicating reverse mode of the exchanger. This increase in matrix Ca^2+^ was sustained for several seconds in the continued presence of InsP_3_ but fell rapidly when cytosolic Ca^2+^ was lowered by perfusion with the Ca^2+^ chelator BAPTA (Fig. 3d; decay half-time of 18.6 ± 2.0 s), indicating the Na^+^–Ca^2+^ exchanger was now in forward mode, transporting Na^+^ into the matrix in exchange for matrix Ca^2+^. CGP-37157 prevented the InsP_3_-induced rise in matrix Ca^2+^ in depolarised mitochondria (Fig. 3d).

In MFN2- and MCU-deficient permeabilised cells bathed in low (1 mM) Na^+^-solution and FCCP and oligomycin to trap the exchanger in reverse mode, matrix Ca^2+^ rose upon exposure to InsP_3_ (Fig. 3e; aggregate data are shown in Fig. 3f) and remained elevated, even after addition of BAPTA to the cytosol (Fig. 3e; decay half-time of 108.2 ± 4.8 s). Because Li^+^ can partially replace Na^+^ in the transport cycles of the exchanger^8^, we added 9 mM Li^+^ to the low Na^+^ cytosolic solution. Stimulation with InsP_3_ led to a rise in matrix Ca^2+^ but perfusion with BAPTA now reduced matrix Ca^2+^ (Fig. 3e; decay half-time of 30.2 ± 2.7 s). The matrix Ca^2+^ rise induced by InsP_3_ in the presence of Li^+^ was prevented by CGP-37157 (Fig. 3e; aggregate data are shown in Fig. 3f). Collectively, these data are consistent with a major role for the exchanger operating in reverse mode to raise matrix Ca^2+^ and then in forward mode to lower it.

To inhibit MCU fully, we used the permeabilised cell system to apply the membrane-impermeable MCU inhibitor ruthenium red. In control experiments, raising cytosolic Ca^2+^ to 10 μM led to a large rise in matrix Ca^2+^ and this was suppressed by ruthenium red (Supplementary Figure 6). Addition of 2 nM ionomycin in 200 nM Ca^2+^ to ruthenium red- and FCCP-treated cells deficient in mitofusin 2 led to the typical small rise in matrix in Ca^2+^ and InsP_3_ evoked a further increase in matrix Ca^2+^ (Fig. 3g). This result confirms the existence of a ruthenium red-insensitive Ca^2+^ uptake activated under depolarised conditions. The Ca^2+^ signal decayed to basal levels when BAPTA was subsequently added to the cytosol (Fig. 3g). As was the case in intact cells, cyclosporine A had no effect on the matrix rise induced by InsP_3_ (Fig. 3g; control pericam ratio increase was 0.076 ± 0.003 and in cyclosporine A it was 0.075 ± 0.002).

### Modelling NCLX activity predicts matrix Ca^2+^ oscillations

Our findings could be replicated by a mathematical model in which the Na^+^–Ca^2+^ exchanger was the only functional Ca^2+^ transporter in the inner membrane of depolarised mitochondria (Fig. 3h, i). We fed into the model experimental data of oscillations in cytosolic Ca^2+^ induced by LTC_4_ following application of 2 nM ionomycin following the protocol used in Fig. 3a, but now obtained from intact cells (Fig. 3h, upper panel). The simulations revealed repetitive fluctuations in exchanger forward and reverse modes (Fig. 3h, middle panel) that led to oscillations in matrix Ca^2+^ (Fig. 3h, lower panel). The oscillatory rise in matrix free Ca^2+^ concentration depended on the amplitude of the cytosolic Ca^2+^ rise. If we fed in the amplitude of measured bulk cytosolic Ca^2+^ rise then the matrix oscillations were small in size (Fig. 3h). Mitochondria sense high local Ca^2+^ from open InsP_3_ receptors at specialised regions where the ER is tethered to mitochondria^15,16^. At these sites, mitochondria are exposed to local cytosolic Ca^2+^ concentrations of tens of μM^1,32^. Cytosolic Ca^2+^ signals of this size led to larger amplitude matrix Ca^2+^ fluctuations through forward and reverse mode Na^+^–Ca^2+^ exchange activity (Fig. 3i). Patch clamp recordings on mitoplasts have reported ~1.5 pA unitary current through the MCU (P_open_ of 0.99) and a whole mitoplast current of ~800 pA, suggesting a single mitoplast expresses ~500 channels^6^. Therefore we simulated matrix Ca^2+^ oscillations for between 1 and 10,000 exchangers. The number of exchangers in Fig. 3h, i were set at 100, with a mitochondrial Ca^**2+**^ buffering capacity of 100 (Fig. 3h) or 1000 (Fig. 3i). The simulations show qualitatively that forward and reverse mode Na^+^–Ca^2+^ exchange is sufficient to generate oscillations in matrix Ca^2+^ that replicate those seen experimentally.

### NCLX activity supports Ca^2+^dependent gene expression

**Fig. 4:**
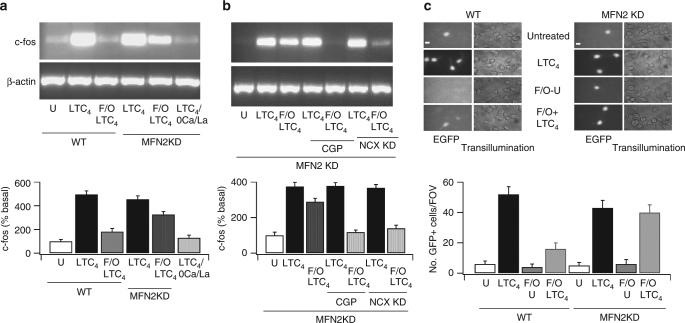
Functional Na^+^–Ca^2+^ exchange supports gene expression in cells with de-energised mitochondria. **a** LTC_4_-induced c-fos gene transcription is reduced by FCCP but rescued following knockdown of mitofusin 2. Lower panel shows aggregate data from three independent experiments. U denotes untreated, F/O FCCP and oligomycin. F/O was applied 5 min prior to stimulation. LTC4/0Ca/La denotes the lack of c-fos response in the absence of Ca^2+^ influx, despite sustained cytosolic Ca^2+^ oscillations^25^. **b** In mitofusin 2-deficient cells, rescue of LTC_4_-induced c-fos transcription in the presence of mitochondrial depolarisation is prevented by either CGP-37157 (labelled CGP) or knockdown of the Na^+^–Ca^2+^ exchanger (NCX KD). **c** NFAT-dependent reporter gene expression is compared for the different conditions shown. The left-hand panel denotes wild-type cells and the right-hand panel responses after knockdown of mitofusin 2. Bar plot in the lower panel denotes aggregate data for the various conditions shown. FOV on *y*-axis is Field Of View. Here, FCCP and oligomycin were applied 5–10 min before stimulation with LTC_4_. Agonist and FCCP/oligomycin were removed after a further 8 min. Scale bar denotes 10 μm. Error bars denote SEM

We asked whether mitochondrial Ca^2+^ import via reverse mode Na^+^–Ca^2+^ exchange was of functional significance. Local Ca^2+^ influx through CRAC channels following leukotriene receptor activation induces Ca^2+^-dependent expression of the immediate early gene c-fos in RBL-1 mast cells^25,33^. Stimulation with LTC_4_ increased c-fos transcription and this was suppressed by mitochondrial depolarisation following pre-exposure to FCCP and oligomycin for 5 min (Fig. 4a; aggregate data in lower panel), an effect that arises from loss of mitochondrial Ca^2+^ buffering of Ca^2+^ entry through CRAC channels and which then leads to enhanced Ca^2+^-dependent slow inactivation of the channels^22,34^. Knockdown of mitofusin 2 had no effect on c-fos expression induced by LTC_4_ but reversed the inhibitory effect of mitochondrial depolarisation (Fig. 4a; aggregate data shown in lower panel). The recovery of c-fos expression in mitofusin 2-deficient cells with depolarised mitochondria was prevented either by exposing cells to CGP-37157 prior to stimulation with LTC_4_ or knocking down the mitochondrial Na^+^–Ca^2+^ exchanger (Fig. 4b).

Ca^2+^ microdomains near CRAC channels activated by LTC_4_ also stimulate the transcription factor NFAT, resulting in expression of a GFP reporter gene driven by an NFAT promoter^35,36^. Stimulation with LTC_4_ induced a substantial increase in the number of GFP-positive cells (Fig. 4c; aggregate data in lower panel) and this was suppressed by brief pre-treatment with FCCP and oligomycin prior to agonist challenge. Knockdown of mitofusin 2 rescued leukotriene receptor-evoked NFAT-driven gene expression in cells with depolarised mitochondria (Fig. 4c).

## Discussion

**Fig. 5:**
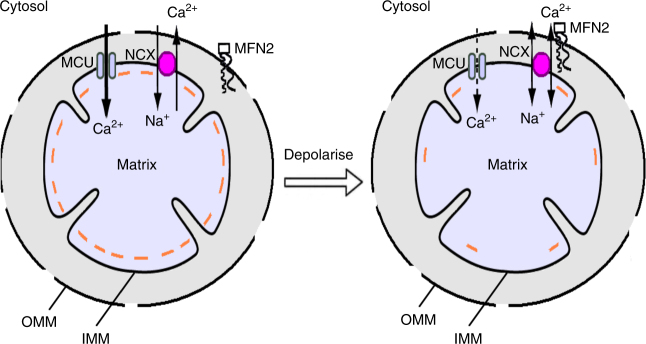
Cartoon summarises Na^+^–Ca^2+^ exchanger transport in mitochondria under the conditions shown The left-hand panel depicts the situation in energised mitochondria. The exchanger operates in forward mode, transporting cytosolic Na^+^ into the matrix in exchange for matrix Ca^2+^, which has entered through the MCU. Mitofusin 2 is located in the OMM and functionally detached from the exchanger. After mitochondrial depolarisation (right-hand panel), the exchanger is now functionally coupled to mitofusin 2 and can operate in both forward and reverse modes. OMM denotes outer mitochondrial membrane, IMM inner mitochondrial membrane

Our results show sequential cycling between reverse and forward transport modes of the mitochondrial Na^+^–Ca^2+^ exchanger results in bidirectional movement of Ca^2+^ across the inner mitochondrial membrane. Bidirectional transport by mitochondrial Na^+^–Ca^2+^ exchange develops in response to physiological levels of receptor stimulation and provides a mechanism whereby cytosolic Ca^2+^ oscillations can faithfully reconvene within an organelle. Reverse mode transport is regulated by mitofusin 2 and requires depolarisation of the mitochondrial membrane potential. A cartoon depicting this is shown in Fig. 5.

The reversal potential of Na^+^–Ca^2+^ exchange is determined by the stoichiometry of ion transport and the Nernst potentials for Na^+^ and Ca^2+^. With an inner mitochondrial membrane potential of ~−180 mV and with typical resting cytosolic and matrix Ca^2+^ and Na^+^ concentrations, the exchanger will operate in forward mode, transporting Na^+^ ions from the cytosol into the matrix in exchange for a Ca^2+^ ion. Depolarisation with FCCP enables the exchanger to operate in both forward and reverse modes, depending on the ambient cytosolic Ca^2+^. FCCP induces a depolarisation of ~100 mV^9^ and so quite marked depolarisation is needed for the exchanger to operate in reverse mode. Depolarisation on this scale is not observed routinely in living cells and reverse Na^+^–Ca^2+^ exchange is unlikely to be of physiological significance for the entire mitochondrial pool. However, in patho-physiological states such as oxidative stress induced by ischaemia or strong local reactive oxygen species production, mitochondrial membrane potential in cardiomyocytes can oscillate or even collapse^37,38^. It is important to note that we found reverse mode Na^+^–Ca^2+^ exchange to be effective only after the combination of mitochondrial depolarisation and knockdown of mitofusin 2. Although mitofusin 2 is widely expressed, it is not expressed uniformly between cells. Muscle has very high levels compared with other cell types. Mitofusin 2 expression is also under control of circulating factors. For example, tumour necrosis factor-α and interleukin-6 both significantly reduce mitofusin 2 expression^39^, suggesting the levels expressed are dynamic. Interestingly, expression of mitofusin 2 also alters in various states^39^. Expression falls significantly in skeletal muscle from type 2 diabetes sufferers as well as in obese patients^39^. Hence it is possible that mitofusin 2 levels might fluctuate sufficiently under certain conditions for the protein to regulate Na^+^–Ca^2+^ exchange activity.

The mechanism whereby mitofusin 2 regulates reverse mode but not forward mode is currently unclear but does not require mitochondrial fusion because knockdown of OPA1 did not replicate the effects of reduction in mitofusin 2. Mitofusin 2 is in the outer mitochondrial membrane and Na^+^–Ca^2+^ exchange is in the inner membrane. It is possible that the two proteins physically interact after mitochondrial depolarisation and this stabilises or enables the reverse mode to operate. Co-immunoprecipitation studies show that myc-tagged Na^+^–Ca^2+^ exchanger is present following pulldown of mitofusin 2 and the amount of exchanger increases in the presence of FCCP (Samanta and Parekh, data presented to reviewers). Reverse mode Na^+^–Ca^2+^ exchanger activity required a small increase in matrix Ca^2+^. This was accomplished by the very low flux through the MCU in depolarised mitochondria or after artificial elevation of matrix Ca^2+^ by a low dose of ionomycin. Whether matrix Ca^2+^ directly binds to the exchanger or its effects are mediated by an intermediary mechanism is currently under investigation.

The mitochondrial Na^+^–Ca^2+^ exchanger, alternating between transport modes generates oscillations in Ca^2+^ within the mitochondrial matrix and these faithfully reflect the cytosolic Ca^2+^ oscillations that trigger exchanger activity. Although the MCU is the main route for mitochondrial Ca^2+^ uptake, our data reveal that the exchanger can, under certain conditions, provide an alternative route for Ca^2+^ entry. More generally, our data show that forward and reverse mode activities of an ion transporter can substitute for a Ca^2+^ channel in generating Ca^2+^ signals evoked by a physiologically relevant agonist.

## Methods

### Cell culture and transfection

Rat basophilic leukaemia (RBL-1) and HEK293 cells were purchased from ATCC and were cultured (37 °C, 5% CO_2_) in Dulbecco’s modified Eagle's medium supplemented with 10% fetal bovine serum, 2 mM l-glutamine and penicillin–streptomycin. RBL cells were transfected with the AMAXA system, using nucleofector cell line kit V solution (from Lonza, Cat. No. VCA-1003) and program T-30 were used. Transfection of HEK293 cells was achieved using the lipofectamine method^35^. For transfection of one dish (10 cm), 50 μl of Opti-MEM was mixed with 4 μl of Lipofectamine 2000 (from Invitrogen, Cat. No. 11668-019) in one eppendorf tube and 50 μl of Opti-MEM was mixed with the required amount of DNA or siRNA in another eppendorf tube. The components were gently mixed by pipetting and after 5 min all the components were mixed together. This transfection mixture was then incubated at room temperature for 20 min to generate lipoplexes for transfection. Thereafter, 1 ml of cell culture medium was added to the transfection mixture, which was then pipetted into the dish. After 1 h incubation at 37 °C and 5% CO_2_, the transfection mixture containing medium was replaced by 2 ml fresh medium. Cells were cultured for 24–48 h in an incubator and then used for experiments.

### Cytosolic Ca^2+^ measurements

Cytosolic Ca^2+^ was measured using the Ca^2+^-sensitive fluorescent dye fura 2. All experiments were conducted at room temperature, using the IMAGO CCD camera-based system from TILL Photonics^40,41^. Cells were incubated with Fura 2-AM (1 μM) for 40 min at room temperature in the dark and then washed several times in standard external solution of composition (in mM): NaCl 145, KCl 2.8, CaCl_2_ 2, MgCl_2_ 2, d-glucose 10, HEPES 10, pH 7.4 with NaOH. After a 15 min de-esterification period, cells were alternately excited at 356 and 380 nm (20-ms exposures) and emission collected >505 nm. Images were acquired every 2 s. Ca^2+^ signals are plotted as R, which denotes the 356/380 nm ratio.

### Cytosolic pH measurements

Cells were incubated with BCECF-AM (5 μg per ml) for 30 min at room temperature in the dark and then washed three times in standard external solution of composition (in mM): NaCl 145, KCl 2.8, CaCl_2_ 2, MgCl_2_ 2, d-glucose 10, HEPES 10, pH 7.4 with NaOH. Cells were alternately excited at 490 and 440 nm (20-ms exposures) and images were acquired every 5 s. The 490/440 nm ratio was calculated and pH signals are plotted as R/R0, where R0 denotes the resting ratio.

### Mitochondrial Ca^2+^ measurements

Matrix Ca^2+^ was measured using the genetically encoded ratiometric pericam protein^21^. Following transfection, with pericam, recordings commenced 24 h later using the TILL Photonics system. Cells were illuminated alternatively at 430 and 488 nm (20 ms exposures) at 0.5 Hz and the emitted light was filtered at >510 nm.

### Mitochondrial membrane potential

Cells were loaded with TMRE (50 nM) in standard external solution for 30 min in the dark, followed by several washes in external solution. Cells were excited at 545 nm and emitted light was collected at >560 nm.

### Cell permeabilisation

Cells expressing pericam were permeabilised by exposure to 5 μM digitonin for 5 min in an intracellular medium containing: 120 mM KCl, 9 mM NaCl, 2 mM KH_2_PO_4_, 10 mM HEPES, 1 mM MgCl_2_, 1 mM Na-pyruvate, 2 mM Mg-ATP, 50 μM EGTA (pH 7.2). Low Na^+^-based intracellular medium contained 129 mM KCl, 2 mM KH_2_PO_4_, 10 mM HEPES, 1 mM MgCl_2_, 1 mM Na-pyruate, 2 mM Mg-ATP, 50 μM EGTA (pH 7.2). Li^+^-based intracellular medium contained: 120 mM KCl, 9 mM LiCl 2 mM KH_2_PO_4_, 10 mM HEPES, 1 mM MgCl_2_, 1 mM Na^+^-pyruvate, 2 mM Mg-ATP, 50 μM EGTA (pH 7.2). In a separate series of experiments, the exposure time for digitionin was determined experimentally by tracking the loss of the fura 2 fluorescence signal at 356 nm from the cytosol after loading with fura 2-AM, as described for cytosolic Ca^2+^ measurements. Intracellular solutions with different Ca^2+^ concentrations (200 nM; 10 μM) were perfused onto the cells as indicated in the text.

### Western blot

Total cell lysates were separated on 10% denaturing sodium dodecyl sulfate polyacrylamide gel electrophoresis. Membranes were blocked with 5% nonfat dry milk in phosphate-buffered saline (PBS) plus 0.1% Tween 20 (PBST) buffer for 1 h at room temperature, were washed with PBST three times and then incubated with primary antibody overnight at 4 °C. ERK2 antibody was from Santa Cruz Biotechnology (Cat. No. sc-154) and was used at a dilution of 1:5000. The antibodies against mitofusin 2 and OPA1 were from Santa Cruz Biotechnology (Cat. No. sc-100560) and BD Transduction Laboratories (Cat. No. 612606) respectively, and used at dilutions of 1:250 and 1:1000. NCLX antibody was kindly provided by Prof. Israel Sekler and was used at 1:1000–1:2000 dilution. The membranes were then washed with PBST again and incubated with 1:2500 dilutions of peroxidase-linked anti-rabbit IgG from Santa Cruz Biotechnology (Cat. No. sc-2004) or anti mouse IgG from BD Bioscience (Cat. No. 554002) for 1 h at room temperature. After washing with PBST, the bands were detected by an enhanced chemiluminescence plus western blotting detection system (Amersham Biosciences). Blots were analysed by UN Scan software.

### RT-PCR and real-time quantitative RT-PCR

After treatment, cells were washed with PBS and total RNA was extracted by using an RNeasy Mini Kit (Qiagen). RNA was quantified spectrophotometrically by absorbance at 260 nm. Total RNA (1 µg) was reverse-transcribed using the iScriptTM cDNA Synthesis Kit (Bio-Rad), according to the manufacturer’s instructions. Following cDNA synthesis, PCR amplification was then performed using BIOX-ACTTM. ShortDNAPolymerase (Bioline) with primers specific for the detection of c-fos and beta-actin (Supplementary Table) were synthesised by Invitrogen. The PCR products were electrophoresed through an agarose gel and visualised by ethidium bromide staining. We performed real-time PCR using an ABI7000 instrument (Applied Biosystems) and detected the fluorescence of samples in 96-well plates by using Taq Man Gene Expression Assays (Applied Biosystems), according to the manufacturer’s instructions. Each 10 μl PCR reaction contained the cDNA, H_2_O, the Master Mix (Applied Biosystems) and Probe & Primer Mix (Applied Biosystems). The mRNA levels of MCU were normalised to GAPDH. Data were analysed by using ABI7000 System Software.

### Gene reporter assay

GFP under an NFAT promoter (gift from Dr Yuri Usachev, University of Iowa) was used as a reporter of Ca^2+^-dependent gene expression. At 24–36 h after transfection with the GFP plasmid, cells were stimulated with LTC_4_ and the % of cells expressing GFP subsequently quantified ~24 h later per field of view^35^. Cells were stimulated with LTC_4_ in culture medium for 8 min and then medium was changed (to remove agonist) and cells were then maintained in the incubator for ~24 h prior to detection of GFP. FCCP/oligomycin was applied 5 min prior to LTC_4_ exposure and was washed out with agonist after 8 min stimulation.

### siRNA knockdown

siRNA against rat mitofusin 2 was from Invitrogen (stealth RNAi^TM^ 5193986). siRNA against human mitofusin 2 was from Origene (Cat No: SR306670). siRNA against OPA1 was from Origene (Cat No: SR505373). siRNA against MCU was from Origene (Cat No: SR508660). siRNA against the rat mitochondrial Na^+^–Ca^2+^ exchanger was from Invitrogen (AACGGCCACUCAACUGUCU) and human mitochondrial Na^+^–Ca^2+^ exchanger was from Origene (Cat No: SR312772). Sequences are listed in the Supplementary Table.

### Mathematical modelling

We denote concentrations with square brackets, e.g. '[Ca^2+^]', and free mitochondrial matrix or cytosolic ion concentrations with the subscripts ‘mito’ or ‘cyt’, respectively. We extended a model of NCX by Kim and Matsuoka^9^ to model the accumulation/depletion of Ca^2+^ within the mitochondrial matrix. The Kim and Matsuoka model predicts the net Ca^2+^ flux in ions per second through a single NCX, and can be written as a single function ‘*f*’:

1 $${\rm Ca}\_{\rm flux} = f\left( {\left[ {{\rm Ca^{2+}}} \right]_{{\rm mito}},\left[ {{\rm Ca^{2 +}}} \right]_{{\rm cyt}},\left[ {{\rm Na^ +}} \right]_{{\rm mito}},\left[ {{\rm Na^ +} } \right]_{{\rm cyt}}} \right).$$

Given a known concentration of Ca^2+^ on the cytosolic side of the mitochondrial membrane ([Ca^2+^]_cyt_) we model the change in concentration of free mitochondrial matrix Ca^2+^ ([Ca^2+^]_mito_) due to a given number of NCX in the mitochondrial membrane. The mitochondria are modelled as having a volume of *V*_mito_ = 1.31 × 10^−15^ l (a cylinder of length 1 μm and radius 0.5 μm with hemispherical caps at each end). We model the mitochondrial matrix as a ‘well mixed’ single compartment, so the change in total mitochondrial [Ca^2+^]_mito_total_ is given by

2 $$\frac{{{\rm d}\left[ {{\rm Ca}^{2 + }} \right]_{{\rm mito\_total}}}}{{{\rm d}t}} = \frac{{N_{{\rm NCX}}\,{\rm Ca\_flux}}}{{V_{{\rm mito}}\,N_{\rm A}}},$$

where *N*_NCX_ is the total number of NCXes in the mitochondrial membrane, and *N*_A_ is Avogadro’s constant. [Ca^2+^]_mito_ is then given by

3 $$\left[ {{\rm Ca}^{2 + }} \right]_{{\rm mito}} = \left[ {{\rm Ca}^{2 + }} \right]_{{\rm mito\_total}}/{\mathrm{buffering}}\;{\mathrm{factor}}.$$

[Ca^2+^]_cyt_ is taken from our fluorescence measurements of cytosolic Ca^2+^ and scaled logarithmically such that the low concentration is 100 nM and the high concentration is either 1.5 or 15 μM (as described in the main text, and shown in Fig. 3g, h). [Na^+^]_mito_ is taken to be 4.54 mM and [Na^+^]_cyt_)as 10 mM. We do not model changes in sodium concentrations as these levels are far above those for [Ca^2+^]_mito_total_ and are assumed to be roughly constant. Mitochondrial membrane potential was set to −20 mV, and temperature to 298 K. The initial condition for [Ca^2+^]_mito_total_ was set to steady state before beginning the simulation. The code to run the simulations shown in Fig. 3 is available as MatLab scripts upon request.

### Statistical analysis

Results are presented as mean ± SEM. Data were compared using Student’s *t*-test or by analysis of variance (ANOVA) for multiple groups. Differences were considered statistically significant at values of *p* < 0.05.

### Data availability

Data supporting the findings of this manuscript are available from the corresponding author upon reasonable request.

## Electronic supplementary material

Supplementary Information

Peer Review File
