# Supplementary material for: Sequential forward and reverse transport of the Na+ Ca2+ exchanger generates Ca2+ oscillations within mitochondria
Source: Nat Commun. 2018 Jan 11;9:156. doi: 10.1038/s41467-017-02638-2 (PMC5765001; doi:10.1038/s41467-017-02638-2)
Supplement: Supplementary file 1 — Supplementary Information [file 41467_2017_2638_MOESM1_ESM.pdf]

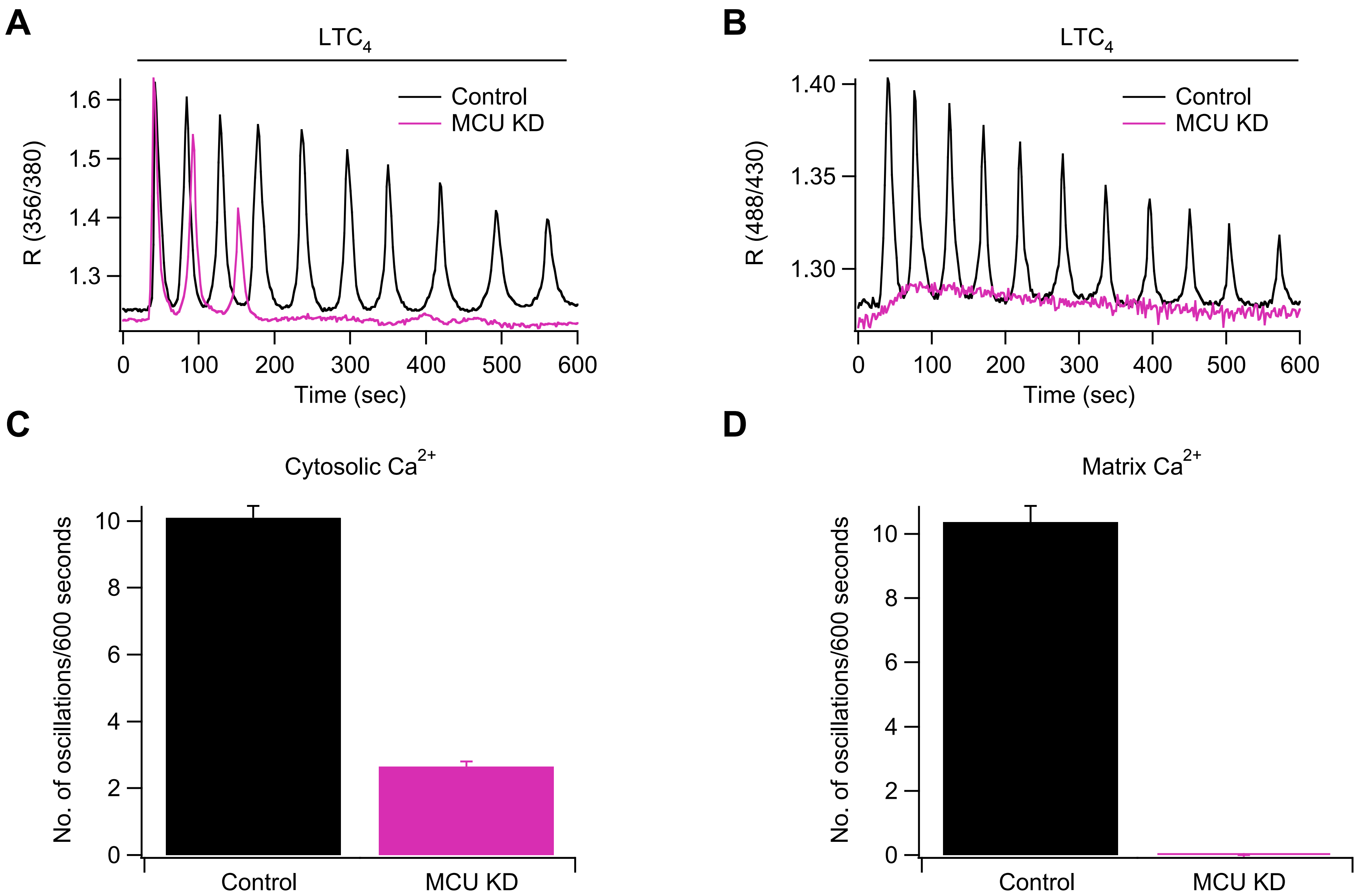

Supplementary Figure 1. MCU knockdown accelerates run down of cytosolic and matrix calcium oscillations in HEK293 cells. **A,** Cytosolic calcium oscillations evoked by LTC4 dissipate quickly after MCU knockdown. **B,** As in panel A, but matrix calcium was measured instead. **C-D,** Aggregate data are summarised. Each bar is the mean of 10-16 cells. Error bars denote SEM.

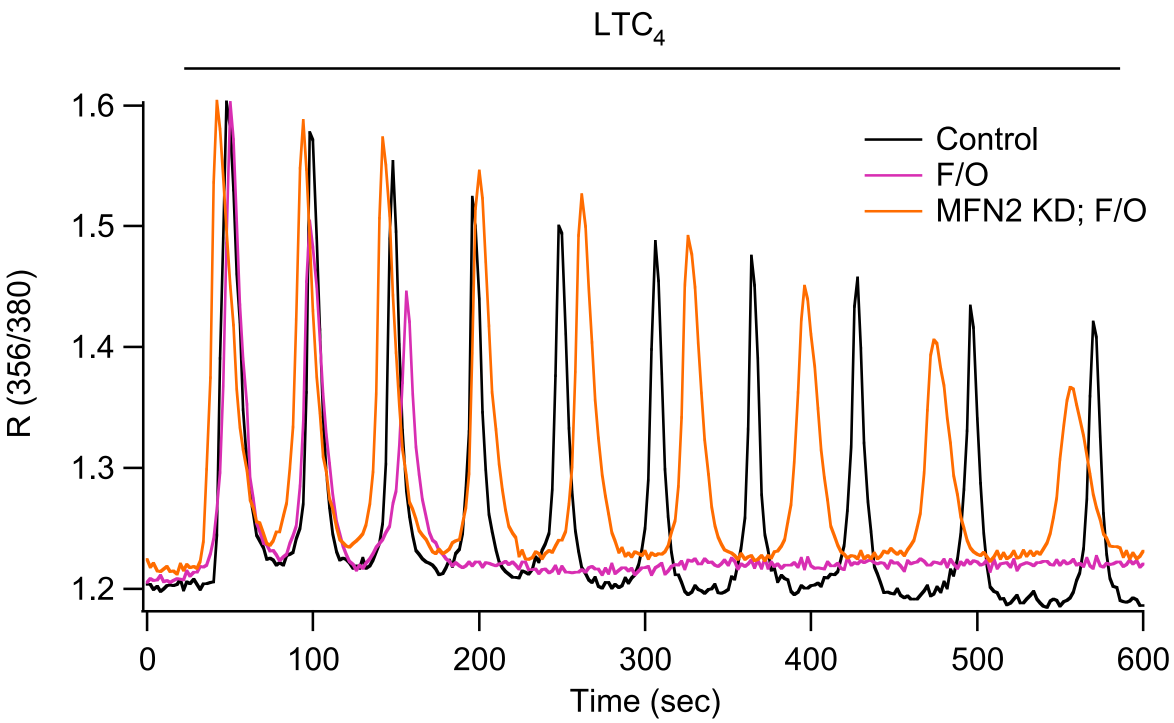

Supplementary Figure 2. Inhibition of cytosolic calcium oscillations in HEK cells by de-energising mitochondria can be rescued by knockdown of mitofusin 2.

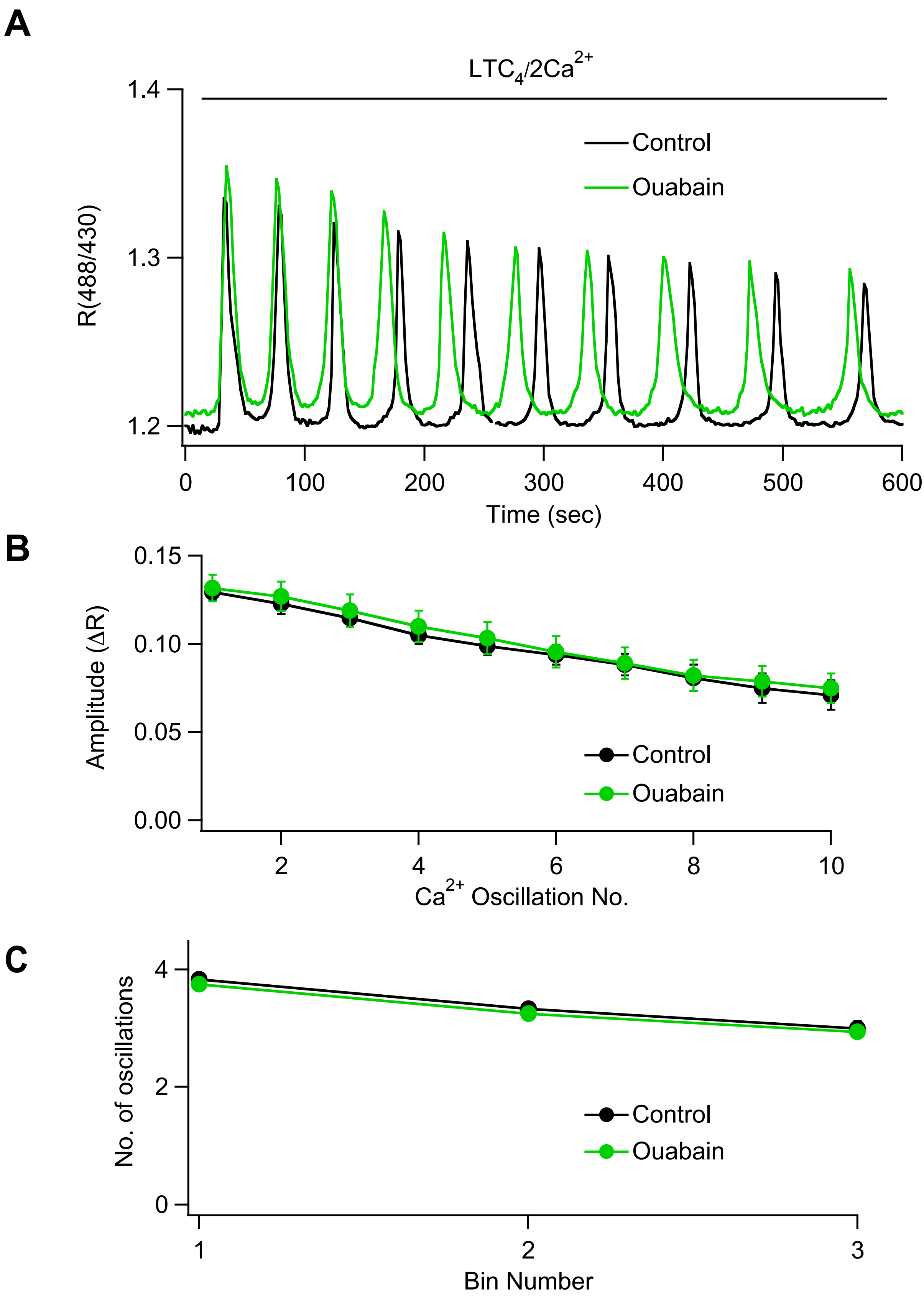

Supplementary Figure 3. Pre-treatment with ouabain (25 μM;15 minutes) does not affect calcium matrix oscillations. **A,** Control recording and one after treatment with ouabain are compared. **B,** Amplitude of each oscillation is compared for the conditions shown. **C,** As in panel B but number of oscillations in each 200 seconds bin are compared. In B and C, each point is the mean of between 17 and 25 cells. Error bars denote SEM.

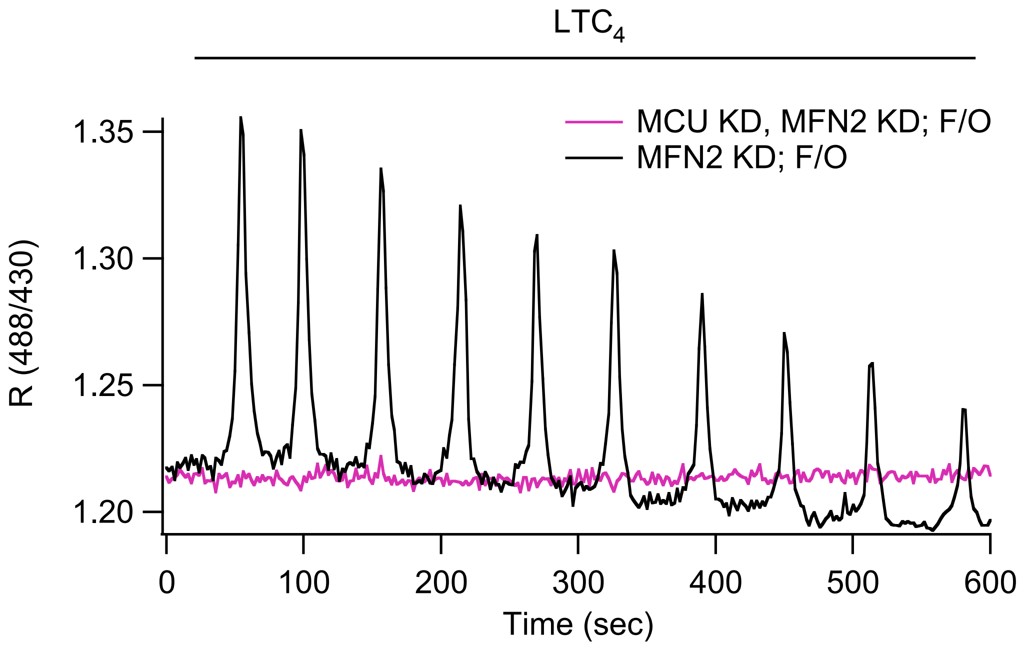

Supplementary Figure 4. MFN2 knockdown fails to rescue matrix calcium oscillations in depolarised mitochondria when MCU levels are reduced. A control recording (after MFN2 knockdown; cell was pre-treated with FCCP and oligomycin for 5 minutes) is shown in black and one in which MCU was also knocked down is shown in red.

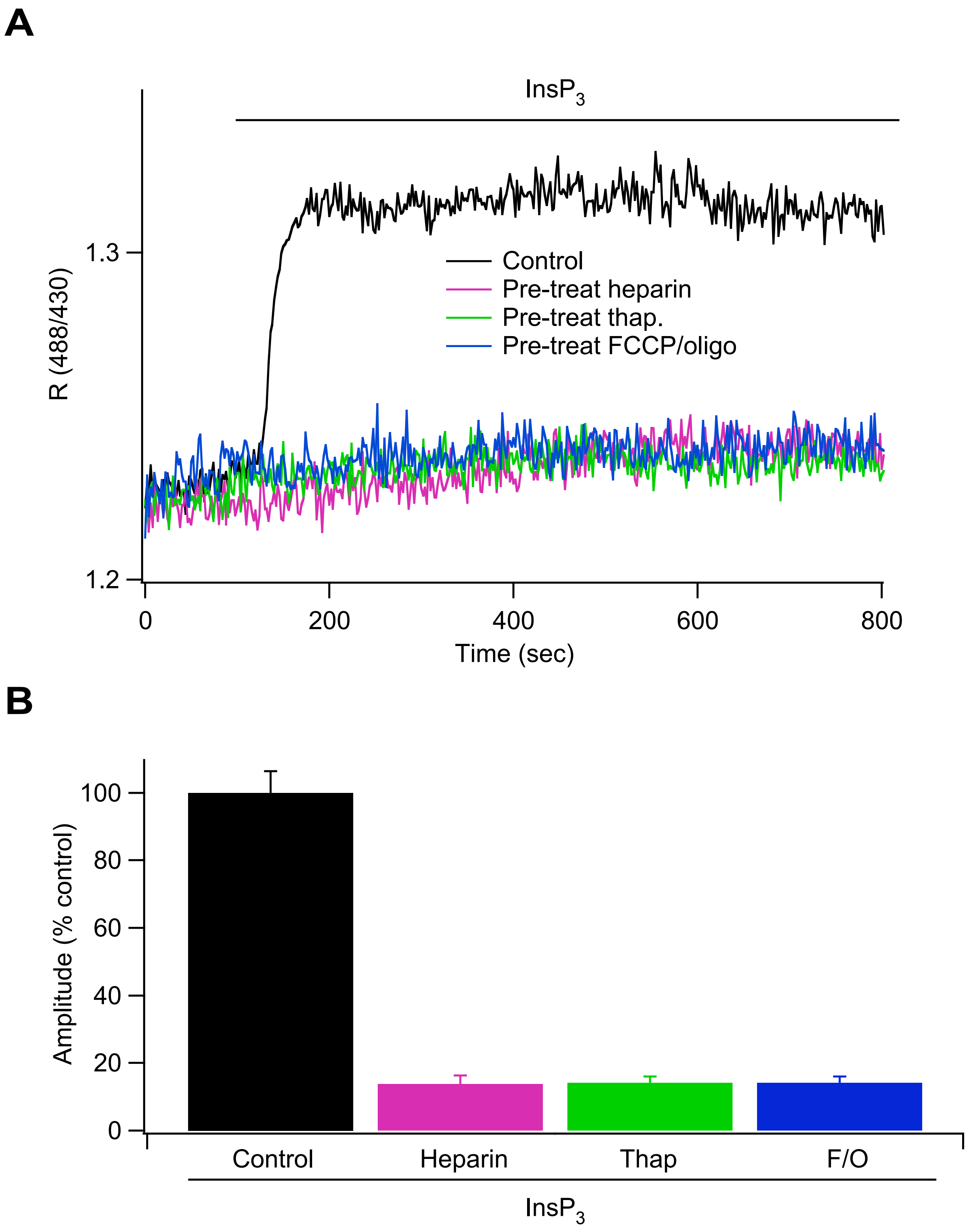

Supplementary Figure 5. Matrix calcium rise in response to $InsP_3$ challenge is due to calcium release from the endoplasmic reticulum. **A,** $InsP_3$ (3 μM) was applied to permeabilised cells in 10 mM sodium-containing cytosolic solution. Pre-treatment with either heparin (400 μg per ml) to block $InsP_3$ receptors or thapsigargin (2 μM) to deplete the store both suppressed the matrix calcium rise to $InsP_3$. Mitochondrial depolarisation with FCCP and oligomycin prevented the matrix calcium rise to $InsP_3$. **B,** Aggregate data are shown. Each bar is between 14 and 21 cells from three separate experiments. Error bars denote SEM.

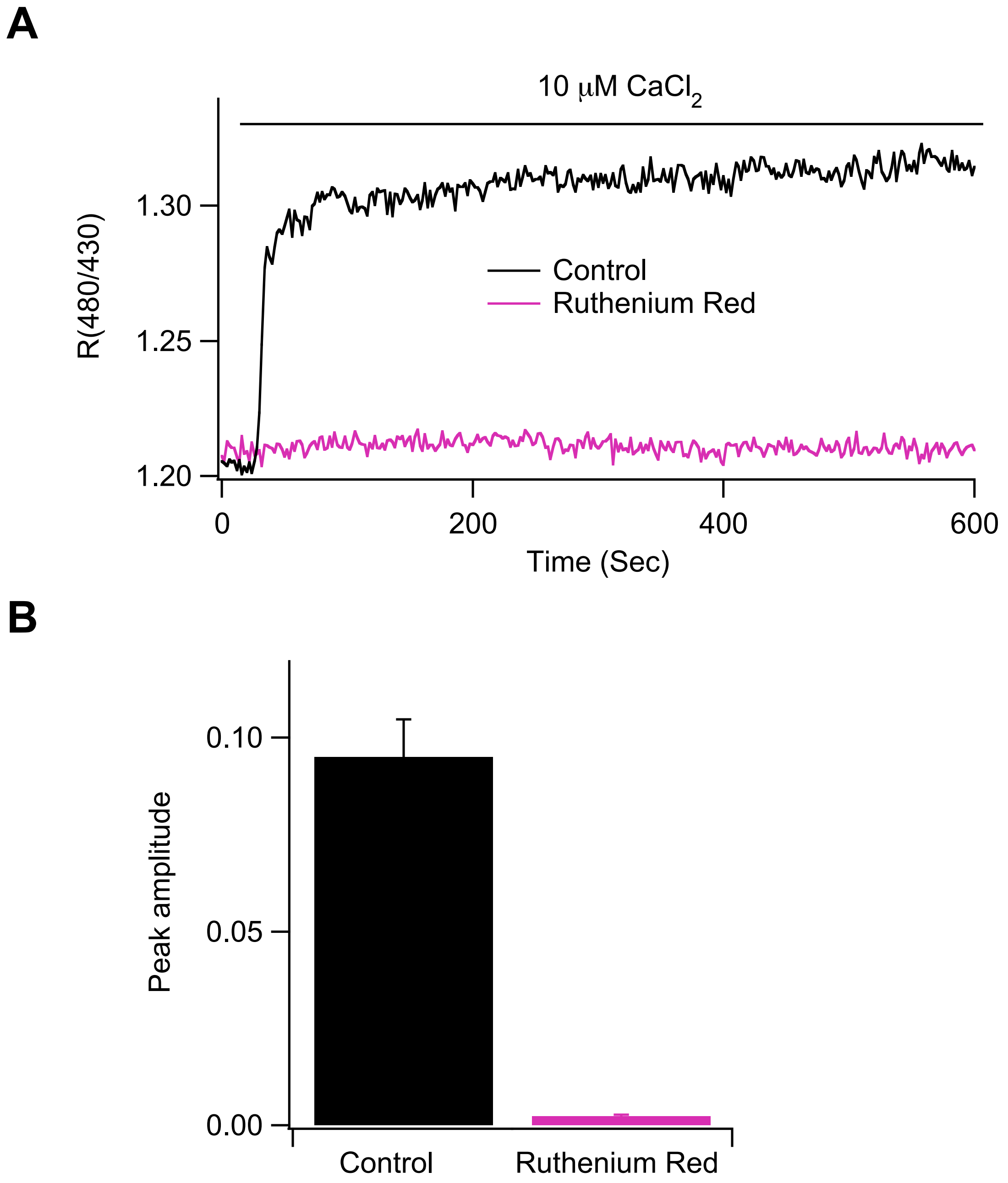

Supplementary Figure 6. Ruthenium Red blocks mitochondrial calcium uptake in permeabilised cells. **A,** Application of 10 μM calcium led to a rise in matrix calcium and this was suppressed by ruthenium red (1μM). **B,** Aggregate data are compared. Each bar is the mean of 12-18 cells from experiments as in panel A. Error bars denote SEM.

A

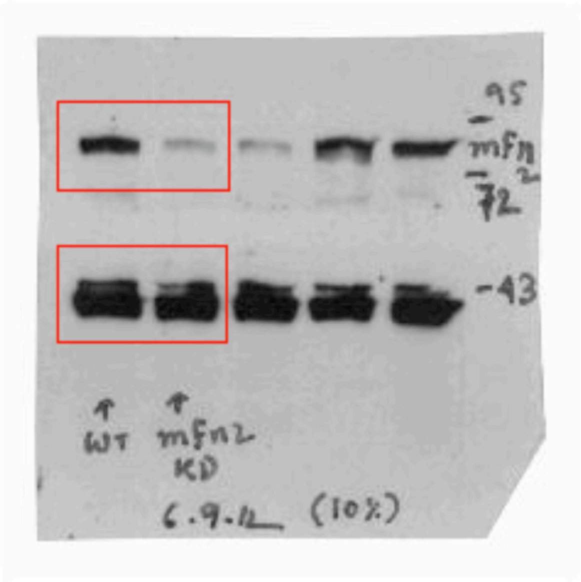

B

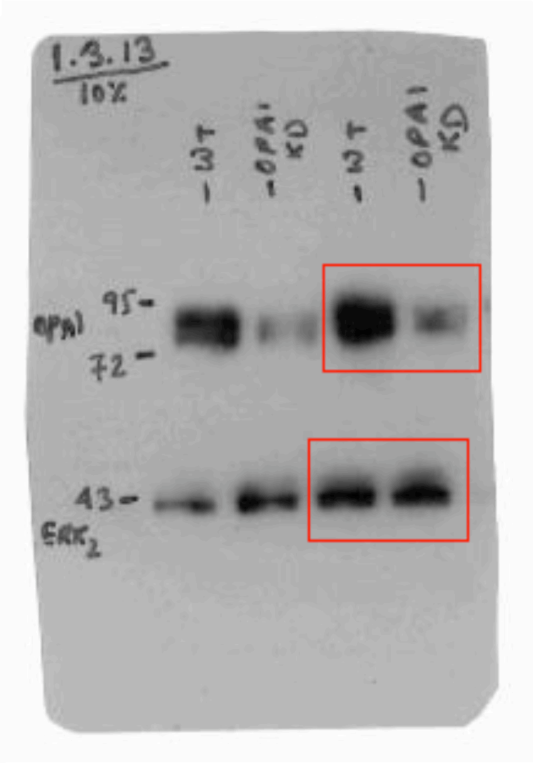

C

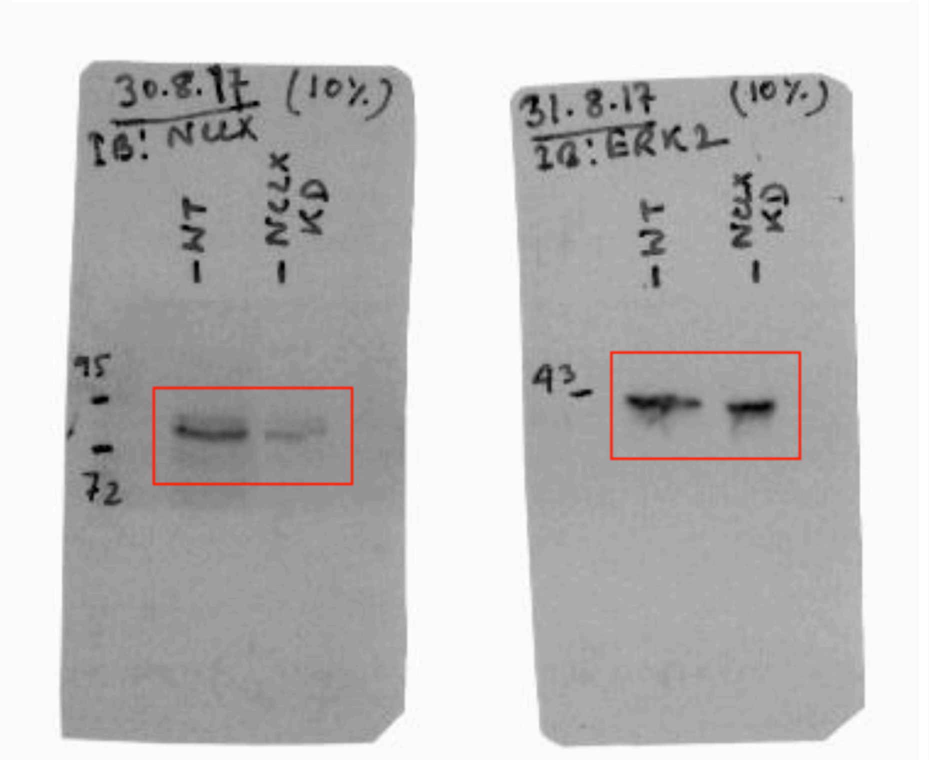

Supplementary Figure 7. Gels used in the paper are shown. **A,** The gel shows knockdown of mitofusin 2 (MFN2), used in Fig. 1I. Upper bands, MFN2; lower bands, ERK2. Lane 1: control, lanes 2&3: MFN2 knockdown, lanes 4&5 control. Lanes 1&2 and 3&4 are different samples. **B,** Knockdown of OPA1. Gel was used in Fig. 1L. Lanes 1&3: control; lanes 2&4 knockdown OPA1. Lanes 1&2 and 3&4 are different samples. **C,** NCLX knockdown. Gel was used in Fig. 2D. Left hand gel is NCLX, right hand gel ERK2.

Primer		Sequence
C-Fos	Forward	5' AGCCGACTCCTTCTCCAGCAT 3'
	Reverse	5' CAGATAGCTGCTCTACTTTGC 3'
β-Actin	Forward	5' TTGTAACCAACTGGGACGATATG 3'
	Reverse	5' GATCTTGATCTTCATGGTGCTAGG 3'

SiRNA	Source	Catalogue No.	Sequence
Rat Mitofusin 2	Invitrogen	Stealth RNAi ™ 5193986	Sequence 1 : CAC UGC AGA CUA UGC AGC AAG ACA U<br>Sequence 2 : AUG UCU UGC UGC AUA GUC UGC AGU G
Human Mitofusin 2	Origene	SR306670	SR306670A - rArGrGrArUrGrUrArGrUrArGrArArGrGrArUrGrGrArUrGGT<br>SR306670B - rGrCrArUrGrGrUrArCrCrArArGrGrArGrUrUrArArGrUrUGA<br>SR306670C - rGrGrUrUrUrArCrUrGrCrGrArGrGrArArArUrGrCrGrUrGAA
Rat OPA1	Origene	SR505373	SR505373A - rArGrCrGrUrUrArArGrArCrArUrGrArArArUrUrGrArArCTC<br>SR505373B - rGrCrCrArGrUrCrCrArArGrCrArGrArArUrArCrArArCrAGA<br>SR505373C - rArGrCrArUrUrUrArGrArGrCrArArCrArGrArUrCrArUrGGA
Rat/human MCU	Origene	SR508660	SR508660A - rCrCrUrArGrArGrArArArUrArCrArArUrCrArArCrUrCrAAG<br>SR508660B - rGrGrCrArGrArArArUrGrGrArUrCrUrUrArArGrArGrArCTG<br>SR508660C - rGrCrCrArGrArGrArCrArGrArCrArArUrArCrUrUrArUrUAT
Rat NCLX	Invitrogen	10620310	AACGGCCACUCAACUGUCU
Human NCLX	Origene	SR312772	SR312772A - rCrCrArGrCrUrCrArGrCrArGrArGrUrCrUrArUrGrGrCrUTC<br>SR312772B - rGrGrArArCrUrGrArGrCrUrArCrUrGrCrArGrArArArGrGGA<br>SR312772C - rCrUrArArUrArCrCrArArCrArGrCrUrArUrGrArCrUrArCGG

SupplementaryTable. Sequences of primers (upper table) and siRNA constructs (lower table) that were used in the study are shown.
